# The Impact of Changes to the Payment Restrictions of the National Reimbursement Drug List in China: An Empirical Study Employing a Stacked Difference-in-Differences Approach

**DOI:** 10.3390/healthcare14193169

**Published:** 2026-09-24

**Authors:** Binbin Chen, Xiaochen Peng, Linlin Cao, Lihua Sun

**Affiliations:** 1 School of Business Administration, Shenyang Pharmaceutical University, No. 103 Wenhua Road, Shenyang 110016, China; 2 Shanghai Health Development Research Center (Shanghai Medical Information Center), No. 181 Xinbei Road, Minhang District, Shanghai 201199, China; 3 School of Pharmaceutical Business, Zhejiang Pharmaceutical University, Ningbo 315500, China

**Keywords:** National Reimbursement Drug List, payment restrictions, volume-for-price, stacked difference-in-differences, pharmacoeconomics

## Abstract

**Background:** Since 2004, China’s National Reimbursement Drug List has implemented a payment restriction management system for certain drugs, to safeguard the fund and avoid unreasonable medication. With the development of medical technology and changes in clinical needs, the fairness and rationality of payment restrictions have come under scrutiny. In recent years, the National Healthcare Security Administration has gradually lifted the payment restrictions for some drugs during dynamic adjustments to the National Reimbursement Drug List; however, the net effect of this policy adjustment still lacks rigorous causal evidence. **Methods:** This study used panel data of 328 drugs experiencing the lifting of payment restrictions from 2018 to 2024 and employed a stacked difference-in-differences method to assess causal effects. The logarithm of annual usage and sales were used as outcome variables. Parallel trend tests were conducted using the event study method, and robustness tests were performed using the Callaway and Sant’Anna estimator. **Results:** Baseline regression showed that after the lifting of payment restrictions, the average annual usage of drugs increased by 60.2% and the average annual sales increased by 180.1%. However, the alternative CS estimator yields large, statistically significant negative estimates, with all estimable cohorts negative (2020: −2.519; 2023: −0.805). The CS estimator automatically excludes the 2024 cohort due to data limitations, while Stacked DID includes all 328 drugs. **Conclusions:** While the evidence suggests that lifting payment restrictions did have an influence on drug utilization and sales, the data do not support a single causal conclusion about whether this influence increases or decreases these outcomes. The divergence between the two estimators reflects differences in sample composition and comparison-group construction: the CS estimator excludes the 2024 cohort, whereas the Stacked DID estimator includes it. Because the two estimates are not computed on a common sample, the divergence cannot be resolved by reweighting; this indicates that the estimated average effect is sensitive to these methodological choices. Policy implications must be conditional on this uncertainty. Cohort-specific CS decomposition reveals that while all cohorts exhibit negative effects, the magnitude decreases a convergence toward positive territory over time.

## 1. Introduction

### 1.1. Background

As a core component of China’s healthcare system, the basic medical insurance system‘s payment policies profoundly influence the supply and utilization of medical services. The National Reimbursement Drug List (NRDL) has undergone several adjustments since its promulgation in 2000. To regulate clinical drug use and safeguard the fund’s security, the 2004 NRDL adjustment began to set payment restrictions for some drugs within the NRDL, such as limiting indications, population groups, or the level of medical institutions. Limiting the payment scope has played a certain role in safeguarding the fund’s security and preventing drug abuse. However, it has also been controversial in its actual implementation, with some arguing that payment restrictions can affect rational clinical drug use and even trigger medical disputes [1]. Since its establishment in 2018, the National Healthcare Security Administration has actively carried out volume-based procurement (VBP) organized by the state, national medical insurance negotiation (Negotiation), and vigorous promotion of payment reforms such as Diagnosis-Related Groups (DRG) and Diagnosis-Intervention Packet (DIP), strengthening its regulatory capacity over the medical insurance fund. Meanwhile, with the rapid development of medical technology and the continuous changes in clinical needs, timely lifting or adjustment of payment restrictions is considered an important measure to maintain fairness in drug access for patients, improve doctor–patient relationships and protect the rights and interests of insured patients. Therefore, in recent years, NRDL dynamic adjustment mechanism has incorporated the adjustment of payment restrictions into its routine work; a large number of drugs have had their original payment restrictions lifted through negotiation or routine adjustments.

From the perspective of classical economic theory, lifting restrictions essentially expands the “market demand” for drugs. Patients who were previously ineligible for reimbursement due to payment restrictions now qualify, leading to a significant increase in drug usage as medical insurance reduces out-of-pocket expenses. Simultaneously, if this increase in volume is not offset by price adjustments, sales revenue will also show an upward trend. This logic aligns with the conclusions of most international research regarding how expanded medical insurance coverage promotes the utilization of healthcare services [2]. From a pharmacoeconomic perspective, payment restriction is a resource allocation mechanism based on a cost-effectiveness threshold. If payment restrictions are indiscriminately lifted, drugs previously restricted due to unclear cost-effectiveness evidence will enter the reimbursement process. This will incur significant opportunity costs, as the fund pays for these low-marginal-value drugs, meaning resources that could be used for more cost-effective interventions are squeezed out. Especially in the absence of a rigorous pharmacoeconomic re-evaluation system, this could lead to unreasonable reimbursement bias for drugs lacking clinical value or products nearing the end of their life cycle, resulting in a net loss of health outcomes. From the perspective of medical insurance fund management, the budgetary shock and moral hazard stemming from the lifting of payment restrictions cannot be ignored. One of the core functions of payment restrictions is to control funding risk by confining fund expenditures within a predictable and manageable range. Lifting payment restrictions means that costs previously borne by patients out-of-pocket or shared by other channels will be transferred in whole or in part to the pooled fund. Without a coordinated mechanism with price adjustments as well as total budget and payment method reforms, this will inevitably create rigid pressure for increased fund expenditures. Moreover, in the principal-agent relationship characterized by information asymmetry in healthcare, the relaxation of payment restrictions may induce a dual moral hazard: suppliers inducing demand and consumers over-consuming. Doctors may inclined to prescribe drugs with more lenient reimbursement rates rather than the most cost-effective options, and patients may become less cost-conscious due to the reduced out-of-pocket expenses.

### 1.2. Literature Review

In recent years, the literature on payment restrictions has largely focused on their impact on drug use and cost control. Numerous empirical studies have evaluated the effectiveness of these restrictions, revealing significant heterogeneity across drug categories. Restrictions on proton pump inhibitors (PPIs), nonsteroidal anti-inflammatory drugs (NSAIDs), and COX-2 inhibitors have shown significant reductions in prescription volume and cost savings in studies from multiple countries [3]. In South Korea, the introduction of clinical guideline-based payment restrictions for diabetes medications led to a decrease in prescriptions for brand-name drugs and combination therapies, an increase in monotherapy, and a slowdown in drug cost growth [4]. However, the effects are not uniform. Restrictions on second-generation antipsychotics, while altering drug choices, failed to reduce overall drug expenditures and even increased the use of other healthcare services [5]. Conversely, studies have also looked at the impact of payment restriction policies on clinical practice, including the misalignment between payment restrictions and clinical guidelines, which may delay effective treatment [6,7]. Administrative delays caused by special authorization procedures are associated with low prescription acquisition rates, increased readmission rates, and increased mortality risks [8,9]. In China, the information technology capabilities and pharmacist intervention effectiveness of hospitals at different levels when implementing payment restriction policies also vary, which may affect the final implementation effect of the policy [10,11]. Specifically, large Class III Level A hospitals have relatively well-developed information systems and can use pre-dispensing prescription review systems to intercept and intervene in real time regarding restricted reimbursement categories, resulting in high policy compliance; in contrast, some primary-care medical institutions, due to a shortage of clinical pharmacists and lagging information systems, experience certain delays and discrepancies when implementing these restriction policies. Recent studies have further examined the evolving landscape of NRDL payment restrictions. Shao et al. (2025) analyzed the current status of payment restriction policies in the 2024 NRDL, finding that while these policies effectively improved healthcare fund utilization efficiency, out-of-pocket costs for some high-priced oncology drugs remained substantial, highlighting the need for integration with multi-level medical security systems [12]. A comprehensive review of the National Drug Price Negotiation policy from 2017 to 2024 found that 553 drugs were incorporated into the NRDL through seven rounds of negotiation, with the policy significantly improving the accessibility and affordability of innovative drugs in China [13].

Existing studies concentrate on the effects of implementing payment restrictions, lacking systematic and rigorous causal inference studies on the important policy adjustment or optimization direction of relaxing or lifting restrictions. The gradual lifting of payment restrictions for drugs in the dynamic adjustment of NRDL has not yet been fully verified regarding its net effects on clinical drug use behavior, patient health outcomes and total medical costs [14], leading to a narrow and incomplete understanding of the payment restriction policy and making it difficult to support dynamic optimization and precise adjustment of the policy. Accordingly, this study assess the potential impact of the policy of lifting payment restrictions, providing a reference for policy adjustment and optimization.

## 2. Materials and Methods

### 2.1. Stacked DID Model

To assess the causal effect of the policy lifting payment restrictions, this study employs a DID model. Since the timing of the lifting of payment restrictions varies for different drugs, traditional two-way fixed effects (TWFE) DID suffers from severe bias when handling such interleaved data. Goodman–Bacon demonstrated that the TWFE estimator is essentially a weighted average of multiple 2 × 2 DID comparisons, where the “treated group as the control for the untreated group” generates a negative weight, causing the estimator to deviate from the true effect and even reverse the sign [15]. Recent methodological guidance on staggered DID designs has emphasized the importance of carefully addressing treatment timing heterogeneity. Freedman (2023) [16] provided practical guidelines for designing DID studies with staggered treatment adoption, highlighting potential problems with conventional estimators when treatment timing varies. Similarly, Zeng et al. (2024) [17] applied a synthetic difference-in-differences approach to evaluate primary healthcare financing reform in rural China, demonstrating the value of quasi-experimental methods for health policy evaluation. The Stacked DID estimator is appropriate as the primary specification when the research question concerns the average treatment effect across all cohorts rather than cohort-specific effects, the treatment timing is staggered with sufficient “not-yet-treated” units available as controls for each cohort, and the parallel trend assumption holds within each cohort-specific subset. Our sample satisfies these conditions, making Stacked DID a suitable choice for estimating the average effect of the restriction-lifting policy across the study period. Therefore, this study uses stacked DID as the baseline analysis method [18]. The core of this method is to construct an independent “analysis subset” for each policy implementation year, with each subset containing only drugs treated in that year (treatment group) and drugs not yet treated in that year (control group), and then stacking all subsets vertically for unified regression. The specific model settings are as follows:

$$\ln\left(Y_{ict}\right)=\alpha_{c}+\lambda_{t}+\beta \cdot \left({Treat}_{i}\times {Post}_{ct}\right)+\varepsilon_{ict}$$

where $\ln\left(Y_{ict}\right)$ represents the usage or sales volume of drug i in subset c in year t; $\alpha_{c}$ represents the stack fixed effects, controlling for the characteristics of each subset not changing over time; $\lambda_{t}$ represents the year fixed effects, controlling for common time shocks; ${Treat}_{i}$ represents the treatment group drugs; ${Post}_{ct}$ represents the period after policy implementation. The coefficient *β* is the average treated effect (ATT) we are interested in. The control group was constructed using an “untreated” strategy: in each subset, drugs that will only be treated after the current year served as the control group. Since the sample includes drugs whose restrictions are lifted in 2024, these drugs serve as “not-yet-treated” controls in the stacks for the 2020–2023 cohorts, providing a sufficient control sample for those cohorts. However, because our data window ends in 2024, we do not observe any post-treatment year for the 2024 cohort, and therefore its own treatment effect cannot be estimated. The 2024 cohort enters the analysis only as a control group in earlier stacks; it is not a treated cohort whose effect is identified.

The model includes stack fixed effects and year fixed effects, with standard errors clustered at the drug level. The stack fixed effects absorb time-invariant differences across cohort-specific subsets, including differences in the composition of drugs; the year fixed effects absorb common time shocks. Because each stack is constructed separately, the fixed effects are nested within stacks, which prevents comparisons across non-comparable cohorts. Each drug-year observation enters the regression with equal weight within its stack; however, because stacks differ in size, stacking does not mechanically assign equal weight to each treatment cohort. We therefore report cohort-specific estimates alongside the pooled estimate, and we do not interpret the pooled coefficient as a simple average of cohort-specific effects. Drug-level fixed effects are not included. Because drugs appear in multiple stacks in different roles, a global drug fixed effect would absorb the treatment indicator for drugs that switch from control to treated across stacks. A specification with drug-by-stack fixed effects would be preferable in principle; we have not estimated this specification, and we identify it as a limitation.

### 2.2. Parallel Trend Test

The validity of DID rests on the assumption of parallel trends: before policy intervention, the trends of outcome variables in the treatment and control groups should be consistent. This paper uses an event study methodology to test this assumption, with the model set as follows:

$$\ln\left(Y_{it}\right)=\sum_{k=-3,k\neq -1}^{3}\beta_{k}\cdot (t-g_{i}=k)+\gamma_{i}+\lambda_{t}+\varepsilon_{it}$$

where $g_{i}$ represents the treatment year for drug i, and k represents the relative treatment year (based on period −1). To further verify the reliability of the conclusions, this paper also uses the Callaway and Sant’Anna estimator for robustness testing. This estimator only uses the “untreated” sample as a control, thus eliminating the negative weighting problem [19].

### 2.3. Data and Information

Data were extracted from public database platforms, including the National Healthcare Security Administration, and the Southern Pharmaceutical Economic Research Institute of the National Medical Products Administration, Menet Data and Yaozhi Data, from 2018 to 2024.

Since the establishment of China’s National Healthcare Security Administration in 2018, the NRDL has been adjusted annually. While a special negotiation for anti-tumor drugs was initiated in 2018, routine adjustments to the NRDL began in 2019, which we therefore treat as the first policy year for evaluation. Our data window spans 2018–2024 (7 years): the year 2018 serves as a pre-treatment baseline, providing at least one pre-policy observation for every drug in the sample. In the NRDL, payment restrictions include limited indications, limited second-line drugs, limited maternity insurance, and limited work-related injury insurance. Between 2018 and 2024, a total of 575 drugs underwent the lifting of restrictions. To maintain data continuity and comparability, this study ultimately included chemical drugs and biologics whose indication restrictions were lifted from the NRDL.

The original sample included 328 drugs covered by the NRDL. These drugs experienced the lifting of payment restrictions in different years during the study period, forming a natural quasi-experimental scenario. Key variables in the data include annual usage (Smallest Dosage Unit), annual sales (RMB), drug type (negotiation list drugs/conventional list drugs), policy type (new indication/lifting of restrictions), and drug price. To mitigate heteroscedasticity and facilitate interpretation, logarithmic values were used for both usage and sales in the regression.

## 3. Results

### 3.1. Parallel Trend Test Results

The sample contains 2296 drug-year observations, involving 328 drugs and spanning a period of 7 years. Figure 1 and Figure 2 illustrate the event study using the logarithm of annual usage and annual sales as outcome variables, respectively.

Most pre-treatment coefficients are statistically indistinguishable from zero, which is consistent with the parallel trends assumption. However, the coefficient at event time −3 for utilization is negative, and its confidence interval does not cross zero. This estimate is based on a small number of drugs observed three years before treatment, and its precision is limited; we therefore interpret the pre-treatment pattern with caution rather than claiming that all pre-treatment coefficients are null. We also note that the post-treatment coefficients fluctuate around a modest positive level rather than following a monotonic upward path, and some post-treatment confidence intervals include zero. This pattern is consistent with a positive but heterogeneous average treatment effect rather than with a uniformly increasing dynamic effect.

The event-study specification is a two-way fixed effects specification, which may be subject to bias under staggered adoption with heterogeneous treatment effects. The estimates reported in Figure 1 and Figure 2 should therefore be interpreted as descriptive evidence on pre-treatment comparability rather than as a fully heterogeneity-robust event study. A fully robust event-study specification would require alternative estimators, which we leave for future work. The stacked DID and CS estimates serve as our primary specifications for the treatment effect.

### 3.2. Benchmark Regression Results

Table 1 reports the baseline regression results based on Stacked DID. Model (1) uses the logarithm of annual usage as the dependent variable, Model (2) uses the logarithm of annual sales revenue as the dependent variable. The results indicate the effect coefficients are significantly positive at the 1% level. Specifically, after the lifting of the payment restrictions, the average annual usage of drugs increased by approximately 60.2%, and the average annual sales revenue increased by approximately 180.1%. This result is consistent with theoretical expectations: the lifting of the payment restrictions releases clinical demand by expanding the reimbursable patient population, and the increase in sales revenue is significantly greater than the increase in usage, indicating that the drug use structure changed after the lifting of restrictions and the use of high-value innovative drugs increased.

### 3.3. Results of Heterogeneity Analysis

Table 2 reports the results of heterogeneity analyses grouped by drug type and policy type, using Stacked DID analysis with usage as the dependent variable. First, the samples were divided into two groups: negotiation list drugs and conventional list drugs. The results showed that the usage effect of negotiation list drugs (0.742*) was greater than that of conventional list drugs (0.308) and significant at the 10% level. The negotiation list drugs are mostly innovative drugs that are urgently needed in clinical practice and have weak substitutability. Before being included in the negotiation list, they usually come with high out-of-pocket expenses, resulting in a high economic burden and medication threshold for patients. Therefore, once the payment restrictions are lifted, the base expansion of the potential beneficiary patient population will be much larger than that of conventional catalogue drugs, showing a higher demand release elasticity. Second, the samples were divided into two more groups: “new indications” and “lift of restrictions” (lifting of population or instruction manual restrictions). The results showed a larger point estimate than the lift-of-restrictions group (0.412), indicating that expanding indications has a much greater pulling effect on clinical use than simply relaxing usage conditions. The approval of a new indication signifies a substantial “expansion” of the drug’s target patient population, resulting in a manifold increase in the number of eligible patients; whereas simply lifting restrictions on the patient population primarily optimizes access to care for existing patients without fundamentally altering the underlying clinical patient base, and thus the resulting incremental effect is relatively limited.

### 3.4. Subgroup Analysis Results

To reveal further formation mechanisms of the policy effect, we conducted subgroup analysis from three dimensions: price changes, market penetration rate before the lifting of restrictions, and implementation batches. The detailed results are shown in Table 3.

#### 3.4.1. The “Volume-for-Price” Effect of Price Adjustments

The price-change subgroups are defined by observed price movements in the post-policy year, which may themselves be endogenous responses to the policy. The following subgroup comparison should therefore be interpreted as descriptive rather than causal. In the reduction group (price reduction > 10%), the usage effect (1.023) was significantly higher than that in the price-stable group (0.292), while the sales revenue effect was opposite between the two groups. The sales revenue of the price-stable group increased significantly, while the sales revenue of the price reduction group did not increase significantly. This comparison clearly reveals the micro-mechanism of “trading volume for price”: accepting a significant price reduction in renewal negotiations in exchange for expanded payment coverage and increased sales volume, with the price reduction partially offsetting the increase in sales revenue. Specifically, in the price-adjustment group, where prices fell by more than 10%, the volume effect (ATT = 1.023) was significantly higher than in the price-stable group (ATT = 0.292), indicating that the price reduction significantly improved affordability and clinical accessibility for patients. At the sales revenue level, however, despite the substantial increase in usage, the sales revenue effect (ATT = 0.681) in the price-reduction group was not statistically significant. These patterns are consistent with a volume-for-price mechanism, but they do not establish that price reductions caused the larger usage increase. A formal test would require product-level price and quantity data.

#### 3.4.2. Differences in Marginal Increments After Removing Constraints

Drugs were divided into two groups based on their median usage in the year prior to the lifting of restrictions: high-base and low-base. The usage effect (0.625) and sales effect (1.387) of the low-base group showed larger point estimates than the high-base group (0.044 and 0.394, respectively). This indicates that drugs with lower market penetration before the lifting of restrictions have greater demand potential, while drugs with high market penetration are nearing saturation, and the marginal increase brought about by the lifting of restrictions is limited. This discrepancy reflects the economic principle of “diminishing marginal utility”: before the policy was implemented, high-penetration drugs had already covered the vast majority of potential demand through out-of-pocket payments or commercial health insurance; the removal of reimbursement restrictions did not create a substantial “incremental number of new patients,” but rather replaced the original out-of-pocket payment method with reimbursement from the medical insurance fund, thereby producing a “substitution reimbursement” effect. Under these circumstances, the incremental costs borne by the health insurance fund did not yield a commensurate increase in health benefits, resulting in a low marginal benefit from the policy investment.

#### 3.4.3. Temporal Heterogeneity of Implementation Batches

The early cohort (2020–2021) showed a negative but insignificant estimate, while the 2023-treated cohort showed a positive estimate. This effect may be related to the strength of policy implementation. In the early stage, only 26 drugs saw lifted restrictions, with the majority among the first batch of drugs negotiated by the national government. Meanwhile, lifted restrictions were often accompanied by drastic price reductions and hospital access barriers, resulting in a short-term failure to respond promptly to demand. With the improvement of policy coordination mechanisms and the adaptation of hospital pharmaceutical management, the implementation of lifted restrictions in the later stages was smoother, and the positive effect became apparent. The early batches (2020–2021) coincided with the initial stages of national medical insurance negotiations and the establishment of the “dual-channel” management mechanism. At that time, hospital pharmaceutical management and drug access processes were not yet fully mature, and some drugs faced implementation obstacles—such as “difficulty gaining hospital access” and “delays in convening pharmaceutical committees”—when entering clinical use, preventing the policy benefits from being promptly passed on to end patients. As the National Healthcare Security Administration has continued to advance monitoring of drug implementation and optimize the access mechanism for designated medical institutions under the medical insurance system, the efficiency of hospital access and clinical acceptance for drugs in the 2023-treated cohort have improved, allowing the policy’s benefits to be fully realized.

### 3.5. Robustness Test Results

To verify the robustness of the baseline results, we used the Callaway and Sant’Anna (CS) estimators for re-estimation. The results show that the ATT of lifting restrictions on the logarithm of annual usage is −1.468, and the ATT on the logarithm of annual sales is −1.108, which is in the opposite direction to the baseline results. Detailed results are shown in Table 4.

The CS estimator and the Stacked DID estimator produce different estimates because they are computed on different comparison bases, not merely because they weight the same cohort-specific effects differently. Specifically, (1) the CS estimator excludes the 2024 cohort entirely, because no post-treatment year is observed for this cohort, whereas the Stacked DID estimator includes the 2024 cohort as a not-yet-treated control group in earlier stacks; and (2) the two estimators construct their control groups differently over time—the CS estimator excludes the 2024 cohort because no post-treatment year is observed for this cohort, so its own treatment effect is not estimated. The 2024 cohort nevertheless contributes control observations in the stacks for earlier cohorts. We also acknowledge a limitation of the current stacked design. In each stack, control drugs contribute observations for all years in the stack window, including years after their own treatment date. We identify it as a priority for future work and refrain from claiming that either estimate is closer to the true effect. These differences in comparison-group construction, rather than differences in weighting, are the likely source of the divergence.

However, the divergence between the two estimators reveals that the estimated average effect is sensitive to the choice of estimator and to differences in sample composition and comparison-group construction. The CS estimator excludes the 2024 cohort; the Stacked DID estimator includes it as a control group. The two estimators also handle control-group transitions differently. Because the two estimates are not computed on a common sample and a common comparison basis, they should not be interpreted as competing estimates of the same parameter. We report the Stacked DID estimate as our primary specification because it addresses our original research question regarding the average effect across all drugs in the sample, while acknowledging that this choice is not neutral and that the CS estimate provides important evidence on the sensitivity of the result. However, the opposite-signed CS estimate constitutes an important source of uncertainty regarding both the magnitude and the direction of the overall average effect. The divergence should not be interpreted as evidence that one estimator is superior to the other; rather, it reflects the inherent difficulty of estimating a single average treatment effect when cohort-specific effects are heterogeneous and differentially weighted by alternative estimators.

The cohort-specific decomposition (Table 5) reveals three important patterns. First, all cohorts exhibit negative CS estimates, which is consistent with the negative overall CS estimate in Table 4. Second, there is a clear temporal gradient: the negative effect is largest for the 2020 cohort (−2.519), remains substantial for the 2022 cohort (−1.599), and is substantially smaller for the 2023 cohort (−0.805). This represents a 68% reduction in the magnitude of the negative effect from 2020 to 2023. Third, the 2021 cohort estimate is not statistically significant (*p* = 0.449), likely due to the very small number of drugs treated in that year. This temporal gradient suggests that policy effects are converging toward positive territory over time, which is consistent with the temporal heterogeneity observed in the Stacked DID subgroup analysis, where recent batches (2023–2024) showed significant positive effects.

It is important to note that the CS estimator automatically excludes cohorts without post-treatment observations. In this study, drugs treated in 2024 have no post-treatment period within the data window (2018–2024) and are therefore excluded from the CS estimation. In contrast, the Stacked DID estimator includes all 328 drugs, with the 2024 cohort contributing as not-yet-treated controls in earlier cohort subsets. This sample difference—rather than any inherent contradiction—contributes to the divergence between the two estimators: the CS estimates are based on a sample that excludes the 2024 cohort, whereas the Stacked DID estimates are based on a sample that includes it as a control group.

## 4. Discussion

The Stacked DID estimates indicate a positive association between the lifting of payment restrictions and drug utilization and sales, suggesting that, conditional on this estimator’s identifying assumptions, the policy may have improved clinical accessibility. However this interpretation is sensitive to estimator choice, and the CS estimates yield opposite conclusions. The rationality of the policy of lifting payment restrictions needs to be examined dialectically from multiple perspectives, including the overall policy effect, potential risks and constraints.

### 4.1. Overall Accessibility Improvement

From the perspective of the policy’s direct objectives, lifting payment restrictions has achieved certain results. First, the clinical accessibility of drugs has significantly improved. The 60% increase in usage indicates a large number of patients who were previously excluded from reimbursement due to payment thresholds have increased medical insurance coverage, especially for drugs with low market penetration, releasing pent-up demand. Second, the price-reduction subgroup shows a larger usage increase (1.023) than the price-stable subgroup (0.292), while the sales revenue coefficient in the price-reduction subgroup is not statistically significant. This pattern is consistent with—but does not by itself demonstrate—a volume-for-price mechanism. A formal test would require product-level data on unit prices and quantities.

### 4.2. Comparison with the Literature

Our findings are broadly consistent with the established literature on the effects of reimbursement policy changes, while also offering novel insights specific to the context of restriction lifting. First, the positive utilization effect we observe echoes the findings of Finkelstein et al. (2012) [2] on the Oregon Health Insurance Experiment, which demonstrated that expanded insurance coverage significantly increases healthcare utilization. However, while the Oregon experiment focused on general healthcare services, our study provides drug-level causal evidence specifically for the pharmaceutical market. Second, compared to studies on the implementation of payment restrictions—such as Green et al. (2010) [3] on PPIs and NSAIDs and Kim et al. (2018) [4] on diabetes medications in South Korea, which documented reduced utilization and cost savings—our findings on the lifting of restrictions represent the mirror image of that policy direction. The magnitude of the sales increase (180.1%) in our study substantially exceeds the cost reductions reported in restriction-implementation studies, suggesting that releasing pent-up demand may generate larger market responses than constraining existing use. Third, unlike Law et al. (2008) [5], who found that prior authorization for antipsychotics failed to reduce overall expenditures due to substitution effects, our heterogeneity analysis reveals that the volume-for-price mechanism can effectively contain cost growth when price reductions accompany the lifting of restrictions, though this condition is not met for the majority (78%) of drugs in our sample. Fourth, the significant difference between new indications and simple restriction lifting in our study provides empirical support for the concerns raised in prior Chinese studies regarding the misalignment between payment restrictions and clinical guidelines. Finally, the temporal heterogeneity we observe—with later implementation batches showing stronger positive effects—parallels the findings of Ackman et al. (2006) [8] and Sheehy et al. (2008) [9] regarding administrative delays, suggesting that policy learning and institutional adaptation over time can substantially improve policy effectiveness.

### 4.3. Potential Risks and Constraints

While the policy of lifting payment restrictions delivers favorable overall effects, it is not unconditional. The potential risks and constraints in the following dimensions determine the boundaries and sustainability of the policy’s effectiveness.

First, the structural pressure on fund expenditures cannot be ignored. Although the “volume-for-price” approach achieved cost control in the price-reduced group, sales in the price-stable group increased significantly, and this group accounts for nearly 78% of the total number of drugs. This means that for most drugs that have not experienced significant price reductions, lifting restrictions will directly translate into fund expenditures. If there is a lack of effective coordination with the overall budget and payment method reforms, the continued expansion of the scope of lifting restrictions may put pressure on the fund balance in the medium to long term.

Second, the heterogeneity of effects reveals the uneven distribution of policy dividends. The usage effect of the high-base group is only 0.044 and not significant, indicating that for drugs already widely used clinically through out-of-pocket or commercial insurance channels, lifting restrictions does not bring significant incremental value. Instead, it may create a “substitute reimbursement” for the existing out-of-pocket market, impacting fund expenditures. Furthermore, the effect of the new indication group is much greater than that of the group with lifted restrictions, indicating that expanding indications has a stronger pull on clinical use. This also means that fund expenditures will flow more towards the expansion of indications for innovative drugs, which needs to be calculated in the budget impact analysis.

Third, there are regulatory challenges related to moral hazards and induced demand. Theoretically, lowering payment thresholds could induce moral hazards on both the supply and demand sides. Doctors might be inclined to prescribe drugs with more lenient reimbursement rates rather than the most cost-effective options, while patients might become less cost-conscious due to lower out-of-pocket expenses. Although the current medical insurance fund supervision system has established multiple lines of defense, such as surprise inspections and intelligent monitoring, the monitoring of the rationality of drug use after lifting restrictions needs to be upgraded simultaneously; otherwise, new waste avenues may result.

### 4.4. The Impact of COVID-19

It is important to acknowledge that our study period (2018–2024) overlaps significantly with the COVID-19 pandemic, which may have affected drug availability and healthcare utilization in general. The pandemic led to disruptions in supply chains, changes in hospital visitation patterns, shifts in prescribing behaviors, and reallocation of healthcare resources toward epidemic response. Our DID framework, with year fixed effects, absorbs common time-varying shocks that affect all drugs uniformly, thereby controlling for the overall impact of the pandemic on the pharmaceutical market. However, the pandemic may have had differential effects across drug categories, regions, and hospital levels that our model does not fully capture. For instance, drugs used for chronic disease management may have experienced utilization declines during lockdown periods, while drugs for acute conditions may have been less affected. Additionally, the accelerated adoption of telemedicine and changes in hospital formularies during the pandemic may have differentially influenced the implementation of payment restriction lifting across drugs and institutions. While our stacked DID design mitigates confounding from time-invariant drug characteristics and common time trends, we cannot entirely rule out the possibility that pandemic-related disruptions differentially affected treatment and control groups. Future research using more granular, region-level data could further disentangle the policy effect from pandemic-related confounding.

### 4.5. Management of Lifting Payment Restrictions Based on Drug Categories

The policy effects of lifting payment restrictions vary significantly across different product categories, price trends, and market penetration stages. Therefore, policy design should adopt a differentiated approach.

First, payment restrictions should be lifted on a differentiated basis according to the type of drug. Heterogeneity analysis shows a larger point estimate than conventional list drugs, and the effect of newly added indications is far greater than that of simply lifting restrictions. It is recommended that in the dynamic adjustment of NRDL, priority should be given to expanding the reimbursement scope of negotiated innovative drugs that are clinically essential and have weak substitutability, especially for newly added indications supported by high-level evidence-based data.

Second, a “volume-price linkage” approach to lifting restrictions should be implemented in conjunction with price changes. Subgroup analysis showed that the volume increase in the price reduction group was significantly higher than that in the price-stable group, but the sales growth was not significant, confirming the effectiveness of the “volume-for-price” mechanism. It is recommended to link the lifting of payment restrictions to drug price negotiations. For companies willing to accept price reductions, the scope of payment can be broadened to achieve a win-win situation of patient accessibility and corporate profits through volume-based price reductions. For drugs with high price rigidity, additional value evidence should be required to demonstrate their cost-effectiveness.

Third, the priority for lifting restrictions should be set based on the market penetration stage. For drugs with low usage before restrictions are lifted, the policy effect is significant, while the effect is not significant for drugs with high usage. This indicates that for drugs that have already achieved high penetration rates through out-of-pocket payments or commercial insurance, lifting payment restrictions mainly generates “substitute reimbursement” rather than incremental health output, resulting in low marginal benefits for the fund. It is recommended to prioritize policy resources for drugs with low market penetration rates, heavy out-of-pocket burdens for patients, and clear clinical value. Lifting restrictions for high-penetration drugs should be coordinated with reductions in payment standards or other cost-control measures to avoid ineffective fund expenditures.

### 4.6. Full-Process Access Management Based on Pharmacoeconomic Evaluation

Reimbursement coverage is the redistribution of limited medical insurance resources. Its rationality highly depends on whether the drugs whose restrictions are lifted possess matching clinical value and economic viability. Therefore, pharmacoeconomic evaluation should be extended from a value judgment tool at the initial access stage to a comprehensive governance tool for dynamic management of reimbursement coverage. Before deciding whether to lift restrictions on a drug, a pharmacoeconomic evaluation should be conducted, focusing on assessing the following dimensions. First, reassessment of the incremental cost-effectiveness ratio (ICER). For drugs applying for lifting of restrictions due to new indications, it is necessary to recalculate whether the ICER after expanding the indications is still within an acceptable threshold range. For drugs that simply lift restrictions on a population or hospital level, it is necessary to assess the marginal change in the impact on the budget caused by the expected increase in the number of patients after the restrictions are lifted. Second, market penetration saturation analysis. For drugs with high usage before the restrictions are lifted, the policy effect is not significant. For varieties that have already achieved widespread clinical use through self-pay or commercial insurance channels, the marginal value of lifting restrictions is limited. Pharmacoeconomic evaluation should combine real-world data to judge the incremental health output and incremental costs brought about by lifting restrictions, avoiding ineffective subsidies to “saturated” markets.

### 4.7. Implications of the Conflicting Robustness Results

The conflicting results between the Stacked DID and CS estimators, along with the cohort-specific CS decomposition, carry several important implications for both methodology and policy interpretation.

First, the divergence does not indicate model failure or invalid identification. Rather, it reflects two fundamental differences between the estimators: (1) the CS estimator excludes the 2024 cohort due to the absence of post-treatment observations, while the Stacked DID estimator includes all 328 drugs; and (2) the two estimators handle control-group transitions differently over time. These differences mean that the two estimates are not computed on a common sample or a common comparison basis. We present both estimates as evidence that the estimated average effect is sensitive to these methodological choices.

Second, the cohort-specific CS decomposition reveals a clear temporal gradient among the estimable cohorts: the negative CS estimates decline in magnitude from −2.519 (2020) to −0.805 (2023). This pattern is consistent with the possibility that the net effect depends on the balance between price reductions and utilization expansion, but it does not establish that later cohorts have positive effects.

Third, the negative CS estimates for sales revenue are consistent with price reductions offsetting volume gains: lower unit prices can reduce total revenue even when physical volume increases. However, this mechanism cannot explain the negative CS estimates for physical utilization. The negative utilization estimates must reflect other features of the CS specification—most importantly, the exclusion of the 2024 cohort and differences in comparison-group construction. We therefore distinguish between the two outcomes: for sales revenue, price reductions provide a plausible partial explanation; for physical utilization, the negative estimates reflect sample composition and estimator choice rather than any direct effect of prices on quantities.

Fourth, the exclusion of the 2024 cohort from the CS estimation means that the two estimators are not computed on the same sample. The CS estimates are based on the 2020–2023 cohorts, while the Stacked DID estimates also include the 2024 cohort as a control group. This sample difference is a source of the divergence between the two estimators, and it also means that the two estimates should not be interpreted as competing estimates of the same parameter.

Fifth, from a policy evaluation perspective, the conflicting results suggest that policy effects are heterogeneous across cohorts. This heterogeneity itself is a meaningful finding, suggesting that the design of restriction-lifting policies should carefully consider the interaction between reimbursement expansion and price negotiation mechanisms. Policymakers should be aware that the overall average effect masks substantial variation across cohorts, and the net effect depends critically on the balance between price reductions and utilization expansion.

This uncertainty has far-reaching implications for policy inference. The current policy recommendations outline potential directions for policy design under the assumption that “stacked DID” estimates reflect average effects.

## 5. Conclusions

This paper evaluates the effect of lifting payment restrictions in China’s National Reimbursement Drug List using stacked DID with CS estimates for robustness. The central finding is not a single causal effect, but estimator sensitivity. The same data, analyzed with two estimators that construct their comparisons differently, produce opposite-signed results.

The Stacked DID estimates—which include all 328 drugs, with the 2024 cohort serving as a not-yet-treated control group—indicate positive associations: usage +60.2%, and sales +180.1%. The heterogeneity analysis suggests that these positive associations are more pronounced for negotiation-list drugs, drugs with newly added indications, drugs with low market penetration, and the 2023-treated cohort. The price-reduction subgroup shows a larger usage increase than the price-stable subgroup, a pattern consistent with a volume-for-price mechanism.

The CS estimates indicate negative effects for all four estimable cohorts, with a decline in magnitude of 68.0% between 2020 and 2023. Because all estimable cohorts are negative, the divergence cannot be resolved by reweighting; it reflects differences in sample composition and comparison-group construction between the two estimators.

The data do not support a single, robust causal conclusion about whether lifting payment restrictions increases or decreases drug utilization and sales. The two estimators are computed on different samples and construct their comparison groups differently, so the divergence cannot be resolved by reweighting the same cohort-specific effects. Any policy recommendations derived from these results are necessarily conditional and require additional evidence—including common-sample comparisons of the two estimators with identical cohorts and follow-up periods—to resolve the empirical uncertainty.

Based on the empirical analysis results and discussion, we propose differentiated policy design by drug category, integration of pharmacoeconomic evaluation into the restriction-lifting decision process, and strengthened post-implementation monitoring to mitigate moral hazard and fund expenditure pressures.

## Figures and Tables

**Figure 1:**
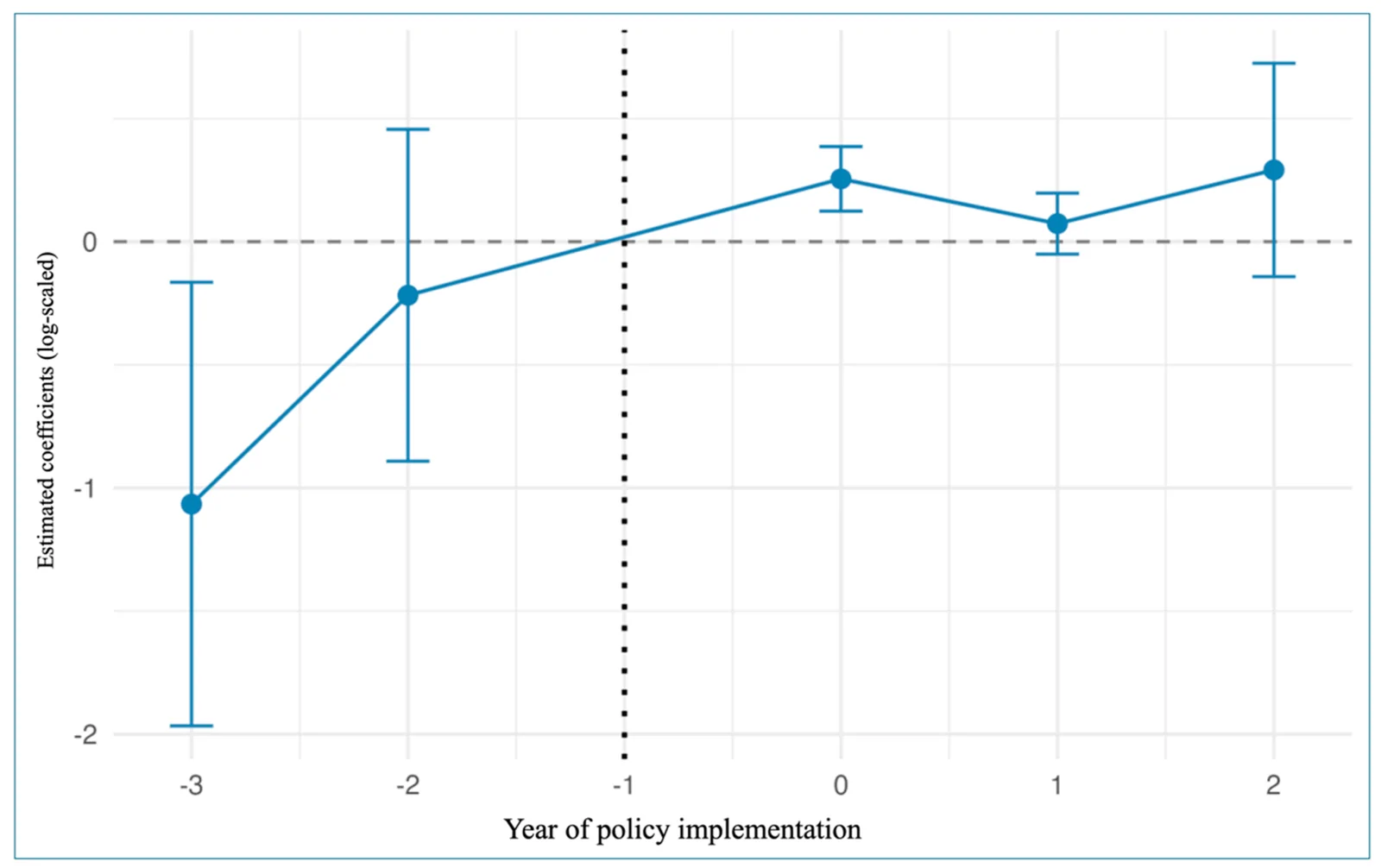
Event study diagram with the logarithm of annual usage as the outcome variable.

**Figure 2:**
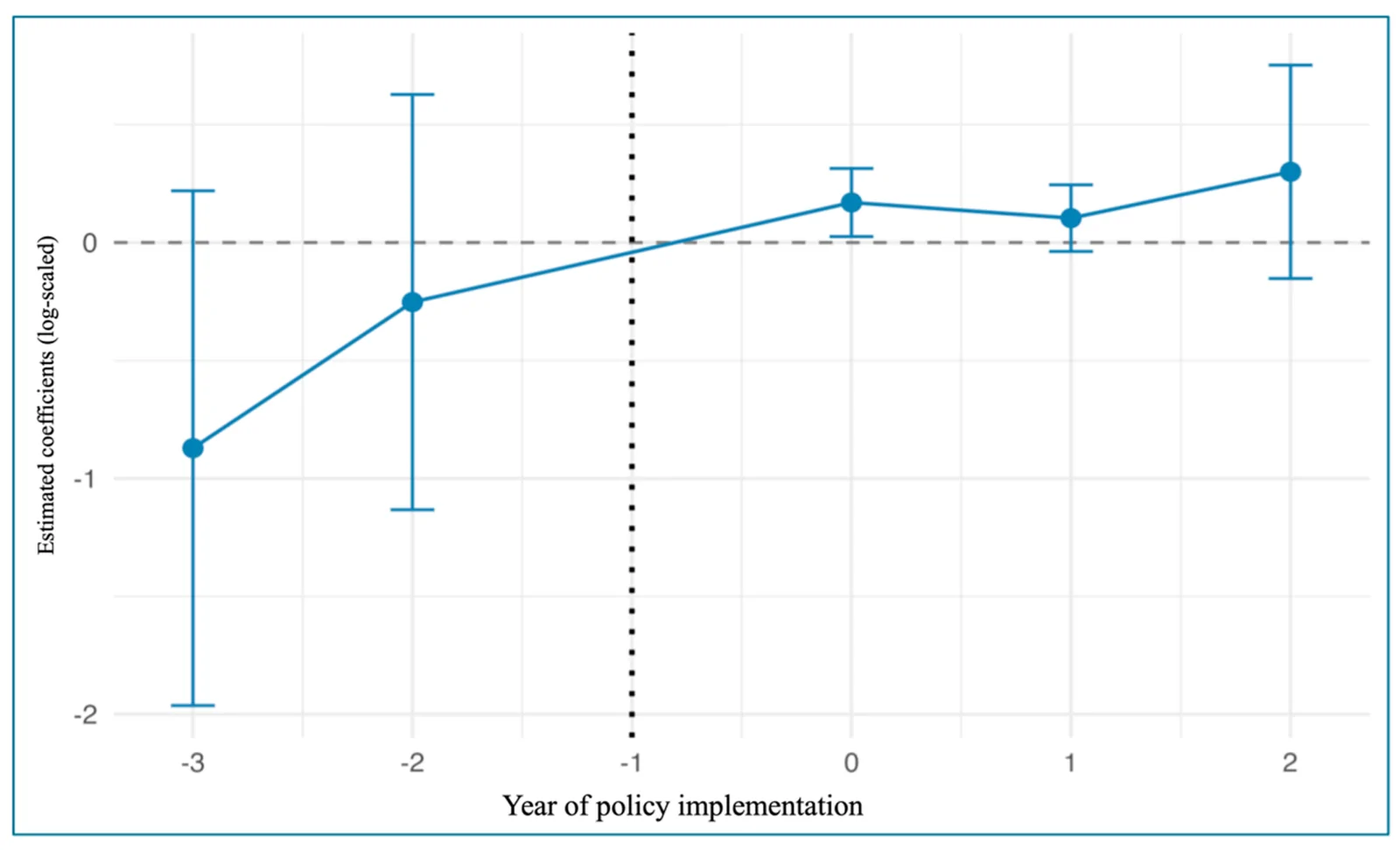
Event study diagram with the logarithm of annual sales as the outcome variable.

**Table 1:** Benchmark regression results.

	Annual Usage	Annual Sales
Processing Group × Policy Follow-up	0.471 **	1.030 ***
	(0.180)	(0.260)
No. Obs.	7879	7879
R2	0.052	0.049
R2 Adj.	0.050	0.048

Note: ** *p* < 0.01, *** *p* < 0.001.

**Table 2:** Results of heterogeneity analysis.

	Negotiation List Drugs	Conventional List Drugs	New Indications	Lift of Restrictions
Processing Group × Policy Follow-up	0.742 +	0.308	1.038 *	0.412 *
	(0.427)	(0.190)	(0.431)	(0.194)
No. Obs.	2103	5744	1441	6438
R2	0.300	0.008	0.295	0.024
R2 Adj.	0.296	0.006	0.290	0.022
R2 Within	0.003	0.001	0.004	0.001
R2 Within Adj.	0.002	0.001	0.003	0.001
AIC	11,550.5	27,717.6	7936.2	32,885.1
BIC	11,618.3	27,797.5	7999.4	32,966.4
RMSE	3.75	2.70	3.77	3.11
FE: cohort_id	X	X	X	X
FE: year	X	X	X	X

Note: + *p* < 0.1, * *p* < 0.05.

**Table 3:** Subgroup analysis results.

Group	Number of Drugs	Observations	Usage ATT	Sales ATT
Full sample	328	7879	0.471 ***	1.03 ***
Auction drug	1	32	Unable to estimate	Unable to estimate
Conventional list drugs	244	5744	0.308	0.782 ***
Negotiation list drugs	83	2103	0.742 +	1.157 **
New indications	53	1441	1.038 **	1.113 **
Lift of restrictions	275	6438	0.412 **	0.834 ***
Price stability	255	6132	0.292	1.061 ***
Price increase (>10%)	11	272	0.591 *	2.254 *
Price reduction (>10%)	59	1400	1.023 **	0.681
Low base	164	3992	0.625 **	1.387 ***
High base	164	3887	0.044	0.394
Mid-term (2022)	121	2541	0	0
Early stage (2020–2021)	26	231	−0.185	−1.089
Recent (2023)	181	5107	0.363 **	0.498 *

Note: + *p* < 0.1, * *p* < 0.05, ** *p* < 0.01, *** *p* < 0.001; three drugs could not be assigned to a price-change category due to missing price information.

**Table 4:** Robustness test results.

Variable	ATT	SE	t-Value
Logarithm of annual usage	−1.4682	0.2293	−6.4036
Logarithm of annual sales	−1.1082	0.2370	−4.6751

**Table 5:** Cohort-specific Callaway and Sant’Anna estimates.

Cohort	Usage ATT	Usage SE	Usage *p*	Sales ATT	Sales SE	Sales *p*
2020	−2.519	0.433	<0.001	−1.804	0.402	<0.001
2021	−0.485	0.641	0.449	−0.373	1.082	0.730
2022	−1.599	0.281	<0.001	−1.145	0.262	<0.001
2023	−0.805	0.225	<0.001	−0.770	0.298	0.010

## Data Availability

The raw data supporting the conclusions of this article will be made available by the authors on request.

## Abbreviations

ATT	Average Treatment Effect on the Treated
CS	Callaway and Sant’Anna
DID	Difference-in-Differences
DIP	Diagnosis-Intervention Packet
DRG	Diagnosis-Related Groups
ICER	Incremental Cost-Effectiveness Ratio
NRDL	National Reimbursement Drug List
TWFE	Two-Way Fixed Effects
VBP	Volume-Based Procurement
